# Inhibitory effect of *Lactobacillus plantarum* and *Lb. fermentum* isolated from the faeces of healthy infants against nonfermentative bacteria causing nosocomial infections

**DOI:** 10.1016/j.nmni.2016.09.003

**Published:** 2016-09-28

**Authors:** M.M. Soltan Dallal, A. Davoodabadi, M. Abdi, M. Hajiabdolbaghi, M.K. Sharifi Yazdi, M. Douraghi, S.M. Tabatabaei Bafghi

**Affiliations:** 1) Food Microbiology Research Center, Tehran University of Medical Sciences, Iran; 2) Division of Food Microbiology, Department of Pathobiology, School of Public Health, Tehran University of Medical Sciences, Iran; 3) Department of Microbiology, Medical School, Babol University of Medical Science, Babol, Iran; 4) Division of Bacteriology, Department of Pathobiology, School of Public Health, Tehran University of Medical Sciences, Iran; 5) Department of Infectious and Tropical Diseases, Imam Khomeini Hospital, Tehran University of Medical Sciences, Iran; 6) Zoonosis Research Center, Tehran University of Medical Sciences, Iran; 7) Department of Medical Laboratory Sciences, School of Para Medicine, Tehran University of Medical Sciences, Tehran, Iran

**Keywords:** Antimicrobial resistance, *Lactobacillus*, nonfermentative bacteria, nosocomial, probiotics

## Abstract

Nosocomial infection constitutes a major public health problem worldwide. Increasing antibiotic resistance of pathogens associated with nosocomial infections has also become a major therapeutic challenge for physicians. Thus, development of alternative treatment protocols, such as the use of probiotics, matters. The aim of this research was to determine the antagonistic properties of *Lactobacillus plantarum* and *Lb. fermentum* isolated from the faeces of healthy infants against nonfermentative bacteria causing nosocomial infections. One hundred five samples of nosocomial infections were collected and processed for bacterial isolation and antimicrobial susceptibility testing following standard bacteriologic techniques. The antibiotic susceptibility test was performed by the disk diffusion method, and antagonistic effect of *Lactobacillus* strains was investigated by well diffusion method. Of 105 samples, a total of 29 bacterial strains were identified as nonfermentative bacteria, including 17 *Acinetobacter baumannii* and 12 *Pseudomonas aeruginosa*. *A. baumannii* showed high resistance to tested antibiotics except ampicillin/sulbactam, and *P. aeruginosa* showed resistance to ampicillin/sulbactam and gentamicin and sensitive to amikacin and meropenem. *Lb. plantarum* had antagonistic properties against both *A. baumannii* and *P. aeruginosa* strains*. Lb. plantarum* had considerable effects on preventing the growth of *A. baumannii* and *P. aeruginosa* strains. However, further research is needed to better understanding of these effects on *P. aeruginosa*.

## Introduction

Nosocomial infections are one of the major problems in hospitals throughout the world. The term “nosocomial” applies to any infections that develop in a patient during a stay in a hospital or other clinical facilities which were not present at the time of admission. It may become clinically apparent either during the hospitalization or after discharge [1]. Nosocomial infections cause morbidity and mortality, functional disability, emotional suffering and economic burden among hospitalized patients [2], [3]. Infections such as surgical wound, respiratory, bloodstream and urinary tract infections are the most common types of nosocomial infections that occur in hospital settings [4]. Nonfermentative bacteria such as *Acinetobacter baumannii* and *Pseudomonas aeruginosa* play an important role in causing nosocomial infections [5].

Probiotics are microorganisms that are believed to provide health benefits for the digestive system when consumed [6]. A number of *Lactobacillus* species, *Bifidobacterium* sp., *Saccharomyces boulardii* and some other microbes have been proposed as and are used as probiotic strains, i.e. live microorganisms as a food supplement to benefit health, such as exerting an antagonistic effect on the gastroenteric pathogens *Clostridium difficile, Campylobacter jejuni, Helicobacter pylori* and rotavirus, neutralizing food mutagens produced in the colon and lowering serum cholesterol [7]. Various health and nutritional effects of lactic acid bacteria have been described, including improvement of the quality of human and animal foods, metabolic stimulation of the synthesis of vitamins and enzymes, stabilization of the intestinal microflora, competence with intestinal pathogens, host innate immune boost, production of antimicrobials, reduction of the risk of colon cancer by neutralizing carcinogens and suppression of tumors by modulating the probiotic strains [8], [9]. Probiotic properties are not seen in all strains but rather are seen only in certain species, depending on the strain [10]. Most bacteria have developed antibiotic resistance; investing in alternative treatments such as probiotics may help solve this problem.

The aim of this research was to study the effect of *Lb. plantarum* and *Lb. rhamnosus* isolated from the faeces of healthy infants in reducing the rates of nonfermentative bacteria causing nosocomial infection.

## Material and Methods

In total 105 bacterial samples were collected from Valiasr Hospital Laboratory, Tehran University of Medical Sciences, Tehran, Iran. These samples were collected from the patients in nosocomial infection epidemics. All the samples were primarily investigated for morphologic and biochemical characteristics, including Gram stain, motility, catalase, oxidation and fermentation, grown at 42°C, indole and esculin test. The isolated strains were transferred into tryptic soy broth after adding 15% glycerol in a 1.5 mL microtube and stored at −20°C. Antimicrobial susceptibility testing was performed by the agar diffusion method on Mueller-Hinton agar as recommended by the United States Clinical and Laboratory Standards Institute (CLSI) [11].

### *Lactobacillus* strains

*Lb. plantarum* 34-5 and *Lb. fermentum* 89-1 were isolated from the faeces of healthy newborns. Identification of these two strains was performed with 16S rDNA gene sequencing. In brief, genomic DNA was extracted according to a previously described method [8]. The PCR primer sequences were as follows: forward primer 5′-CTCGTTGCGGGACTTAA-3′, and reverse primer 5′-GCAGCAGTAGGGAATCTTC-3′ (Bioneer, Korea). The reaction mixture consisted of 3 pmol primers, 1.5 mM MgCl_2_, 0.2 mM dNTPs, 2 μL of genomic DNA, 5 μL 10× PCR buffer and 1.5 U of Taq DNA polymerase (Sinaclon, Iran) in a final volume of 50 μL. The PCR program started with an initial denaturation at 94°C for 2 minutes, followed by 30 cycles of 94°C for 30 seconds, 53°C for 1 minute and 72°C for 1 minute [12]. PCR products were separated by agarose gel electrophoresis (1.5% w/v) and visualized by staining with ethidium bromide. The PCR products of strains were sent to a sequencing company (Bioneer, Korea), and the 16S rDNA sequences were compared to known sequences in GenBank using the Basic Local Alignment Search Tool (BLAST; http://www.ncbi.nlm.nih.gov/blast).

### Antagonistic test

To test the antagonistic effect of *Lactobacillus,* the nonfermentative isolates were first cultured on MacConkey and then on nutrient agar. The *Lactobacillus* strains were inoculated into de Man, Rogosa and Sharpe (MRS) broth and incubated in anaerobic jar at 37°C for 24 hours. On the surface of nutrient agar plate, holes 5 mm in diameter and depth were created under sterile conditions using a Pasteur pipette. Then, using a sterile swab, nonfermentative bacteria of 1/10 McFarland dilutions were inoculated into the surface of nutrient agar. The MRS broth containing *Lactobacillus* was centrifuged at 6000 rpm for 10 minutes. Supernatant was filtered with a bacteriologic filter. Then 100 μL of solution of each of lactobacilli was poured into a separate well. The media were kept in the refrigerator for 2 hours until the liquid was absorbed, then transferred into the incubator and incubated for 14 to 15 hours at 37°C. After incubation, the diameter of the inhibition zones (mm) around the well was measured using a ruler. The antagonistic effect of lactobacillus against nonfermentative isolates was interpreted on the bases of inhibitory growth zones as follows: negative (−), <11 mm; medium (+), 11 to 16 mm; strong (++), 17 to 22 mm; and very strong (+++), >22 mm [9]. Standard *Lb. rhamnosus* GG, obtained from the Department of Microbiology, School of Health, Tehran University of Medical Sciences, was used as a control.

### Statistical analysis

Data of each assay were analysed by one-way analysis of variance by SAS 9.2 software (SAS Institute, Cary, NC, USA). Comparison among treatment means was performed using Duncan’s new multiple range test. Differences were considered significant at *P* < 0.05.

## Results

**Fig. 1:**
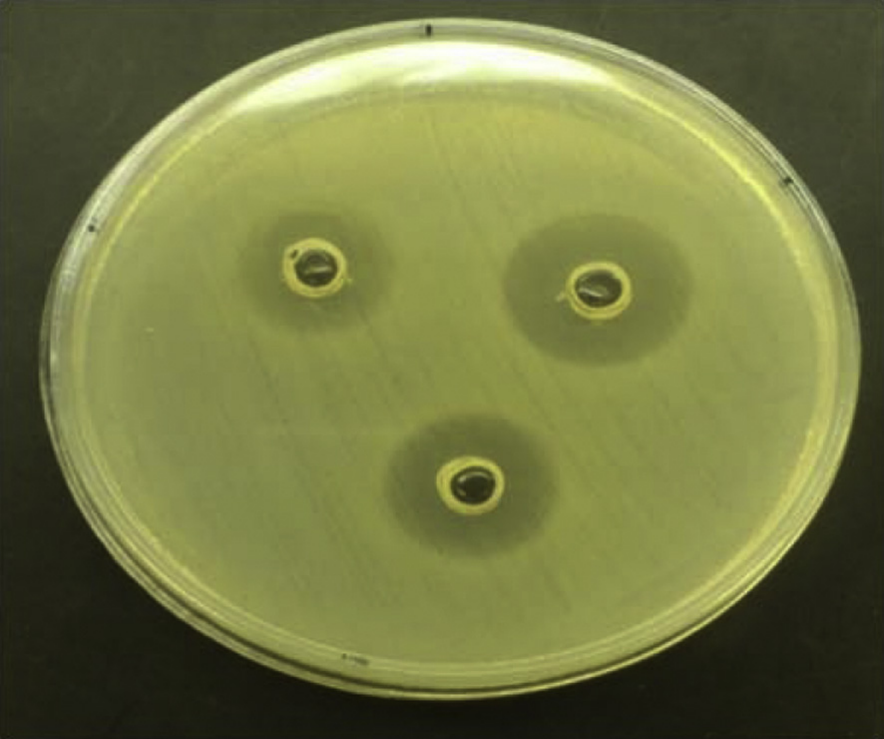
Inhibition zone of *Acinetobacter baumannii* caused by *Lactobacillus* spp. (Top left) *Lb. fermentum* 89-1 (16 mm). (Top right) Inhibition zone of *A. baumannii* caused *by Lb. plantarum* 34-5 (19 mm). (Bottom) *Lb. rhamnosus* GG (17 mm).

**Fig. 2:**
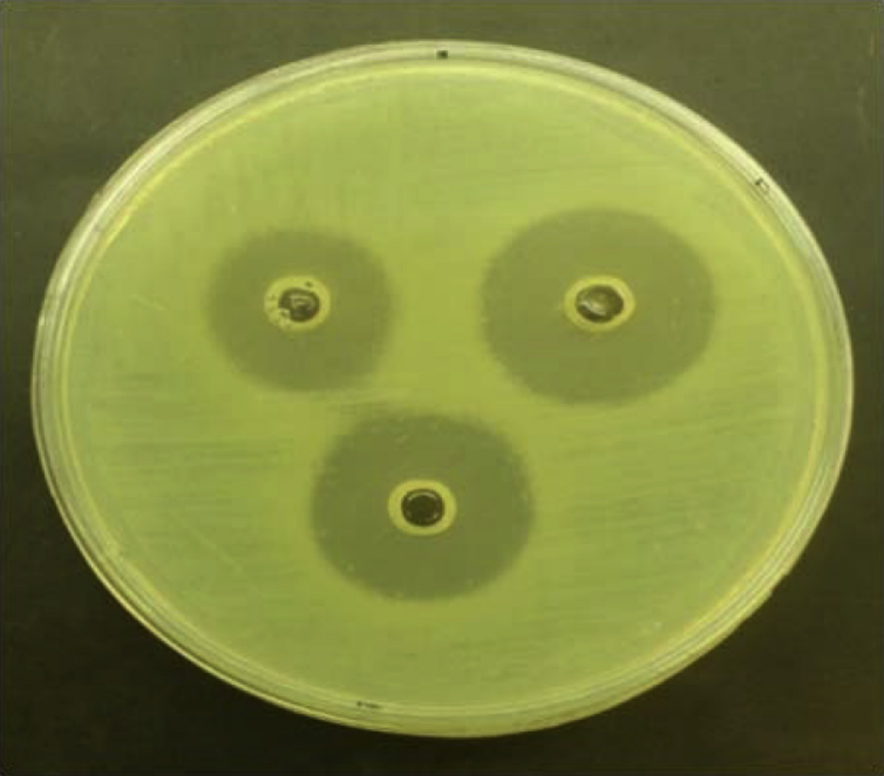
Inhibition zone of *P. aeruginosa* caused by *Lactobacillus* spp. (Top left) *Lb. fermentum* 89-1 (20 mm). (Top right) Inhibition zone of *P. aeruginosa* caused by *Lb. plantarum* 34-5 (24 mm). (Bottom) *Lb. rhamnosus* GG (22 mm).

Of 105 samples, a total of 29 were identified as nonfermentative bacteria, including 17 *A. baumannii* and 12 *P. aeruginosa*. Table 1 shows the antimicrobial susceptibility patterns of *A. baumannii*. High resistance to tested antibiotics was seen except ampicillin/sulbactam, and 100% resistance to cotrimoxazole was observed. *P. aeruginosa* showed resistance to ampicillin/sulbactam and gentamicin and was sensitive to amikacin and meropenem (Table 2, Table 3). *Lb. plantarum* and *Lb. rhamnosus* had antagonistic properties on both *A. baumannii* and *P. aeruginosa* (Table 4). All the tested *Lactobacillus* strains had a significant antagonistic effect on *A. baumannii. Lb. plantarum* 34-5 had a more powerful effect compared to the other lactobacilli (Table 4). All the tested lactobacilli isolates had a significant antagonistic effect on *P. aeruginosa*. *Lb. plantarum* 34-5 had more powerful effect compared to the other lactobacilli (Table 5). The inhibition zones of *A. baumannii* and *P. aeruginosa* isolates by lactobacilli are shown in Fig. 1, Fig. 2.

## Discussion

Eradication and treatment of infections caused by *P. aeruginosa* and *A. baumannii* are difficult because of their high resistance to antibiotics and disinfectants [13], [14]. These bacteria can cause serious infections in hospitalized patients because they can grow under a variety of different conditions and have acquired widespread antibiotic resistance. They therefore result in important nosocomial infections that impose high costs on healthcare [15]. Lactobacilli are harmless microorganisms capable of producing acid secretion, bacteriocins and other by-products that can neutralize some pathogens, can regulate the inflammatory response of the immune system and can be used in the treatment of gastrointestinal disorders [16], [17].

Our results showed that lactobacilli had an inhibition growth effect on both *A. baumannii* and *P. aeruginosa*. These results are in agreement with previously reported findings that different strains of lactobacilli inhibit the growth of bacteria such as *Staphylococcus aureus, Escherichia coli, P. aeruginosa, Klebsiella pneumoniae,* and *Burkholderia cepacia* [18], [19]. Others have reported that lactobacilli had an inhibitory effect on the growth of both Gram-negative and Gram-positive bacteria [20]. In addition, there is also *in vitro* report of probiotics against pathogenic bacteria [21]. Several previous studies have shown that probiotic production factors other than lactic acid, such as bacteriocins, proteinase, peroxidase and exopolysaccharide, can exert antibacterial effects [18]. Some studies have reported that lactobacilli such as *Lb. plantarum, Lb. paracasei, Lb. fermentum, Lb. bokash* and *Lb. boots* isolated from the faeces of infants had inhibitory activity against food-contaminated bacteria such as *E. coli, S. aureus, Yersinia enterocolitica* and *Bacillus cereus* [9], [22]. A previous study demonstrated the antibacterial effect of *Lactobacillus* isolated from breast milk against the gastrointestinal pathogenic bacteria *E. coli, Shigella, Pseudomonas* and *Salmonella* [22]. Another study found that strains of lactobacilli lower the effect of production of elastase and biofilm formation [15].

## Conclusion

The lactobacilli had good effects on preventing the growth of *A. baumannii and P. aeruginosa*. These results are in agreement with other published reports from different countries that indicate that infection control efforts may be achieved with probiotic bacteria. We believe that more attention should be paid to these areas, particularly to create a standardized approach.

## Figures and Tables

**Table 1:** Antimicrobial resistance patterns of *Acinetobacter baumannii* isolates

Antibiotic	Resistant abundance, n (%)	Intermediate abundance, n (%)	Sensitive abundance, n (%)
Amikacin, 30 μg	13 (76.47)	1 (5.88)	3 (17.64)
Ciprofloxacin, 5 μg	16 (94.11)	1 (5.88)	—
Piperacillin/tazobactam, 100/10 μg	14 (82.35)	2 (11.76)	1 (5.88)
Cotrimoxazole, 25 μg	17 (100)	—	—
Meropenem, 10 μg	15 (88.23)	—	2 (11.76)
Ampicillin/sulbactam, 10/10 μg	8 (47.05)	1 (5.88)	8 (47.05)
Ceftriaxone, 30 μg	17 (100)	—	—
Ceftazidime, 30 μg	15 (88.23)	—	2 (11.76)

**Table 2:** Antimicrobial resistance patterns of *Pseudomonas aeruginosa* isolates

Antibiotic	Resistant abundance, n (%)	Intermediate abundance, n (%)	Sensitive abundance, n (%)
Amikacin, 30 μg	2 (16.66)	3 (25)	7 (58.33)
Ciprofloxacin, 5 μg	5 (41.66)	2 (16.66)	5 (41.66)
Piperacillin/tazobactam, 100/10 μg	6 (50)	—	6 (50)
Gentamycin, 10 μg	9 (75)	—	3 (25)
Meropenem, 10 μg	6 (50)	—	6 (50)
Ampicillin/sulbactam, 10/10 μg	11 (91.66)	—	1 (8.33)
Ceftriaxone, 30 μg	7 (58.33)	1 (8.33)	4 (33.33)
Ceftazidime, 30 μg	7 (58.33)	—	5 (41.66)

**Table 3:** Inhibition zone in susceptibility testing of *Acinetobacter baumannii* and *Pseudomonas aeruginosa* (mm)

Isolate	Ceftazidime 30 μg	Ceftriaxone 30 μg	Ampicillin/sulbactam 10/10 μg	Meropenem 10 μg	Gentamicin 10 μg	Cotrimoxazole 25 μg	Piperacillin/tazobactam 100/10 μg	Ciprofloxacin 5 μg	Amikacin 30 μg
1	11	7	16	9	NA	8	12	10	13
2	10	8	17	9	NA	8	11	10	12
3	12	8	17	11	NA	7	12	11	12
4	12	6	10	12	NA	9	18	9	9
5	11	5	9	9	NA	8	13	11	13
6	10	6	16	11	NA	9	12	13	14
7	18	6	17	6	NA	10	19	10	15
8	13	7	17	6	NA	5	21	12	11
9	10	5	10	6	NA	9	14	10	17
10	11	6	9	10	NA	6	15	8	18
11	10	7	7	9	NA	5	12	10	14
12	10	8	9	8	NA	5	11	11	12
13	19	6	11	7	NA	8	14	16	13
14	9	5	8	8	NA	7	13	11	11
15	12	5	16	16	NA	7	12	12	10
16	13	6	18	17	NA	5	11	10	18
17	11	9	7	9	NA	6	14	10	9
18	14	12	8	15	11	NA	14	17	18
19	13	10	9	13	10	NA	13	13	19
20	13	10	8	20	8	NA	14	14	12
21	19	15	7	13	8	NA	21	21	21
22	19	21	6	19	10	NA	21	22	19
23	18	21	15	22	15	NA	22	18	20
24	12	10	7	14	9	NA	23	15	15
25	11	11	9	14	8	NA	14	21	18
26	19	13	10	19	7	NA	13	14	15
27	13	22	9	20	17	NA	13	22	14
28	11	21	8	21	17	NA	21	14	16
29	11	11	8	13	9	NA	22	22	18

NA, not applicable.

**Table 4:** Antagonistic effect (inhibition zone) of *Lactobacillus fermentum* 89-1, *Lb. plantarum* 34-5 and *Lb. rhamnosus* GG against *Acinetobacter baumannii* isolates

*Lactobacillus* genus	Antagonistic effect (inhibition zone)^a^ by abundance, n (%)			
	−	+	++	+++
*plantarum* 34-5	—	7 (41.17)	10 (58.82)	—
*fermentum* 89-1	—	14 (82.35)	3 (17.64)	—
*rhamnosus* GG	—	15 (88.23)	2 (11.76)	—

a Inhibitory growth zones were interpreted as follows: negative (−), <11 mm; medium (+), 11–16 mm; strong (++), 17–22 mm; and very strong (+++), >22 mm.

**Table 5:** Antagonistic effect (inhibition zone) of *Lactobacillus fermentum* 89-1, *Lb. plantarum* 34-5 and *Lb. rhamnosus* GG against *Pseudomonas aeruginosa* isolates

*Lactobacillus* genus	Antagonistic effect (inhibition zone)^a^ by abundance, n (%)			
	−	+	++	+++
*plantarum* 34-5	1 (8.33)	—	10 (83.33)	1 (8.33)
*fermentum* 89-1	1 (8.33)	9 (75)	2 (16.66)	—
*rhamnosus* GG	1 (8.33)	3 (25)	8 (66.66)	—

a Inhibitory growth zones were interpreted as follows: negative (−), <11 mm; medium (+), 11–16 mm; strong (++), 17–22 mm; and very strong (+++), >22 mm.
